# The volatile organic compounds and palatability of mixed ensilage of marigold (*Tagetes erecta* L.) crop residues

**DOI:** 10.1038/s41598-023-28511-5

**Published:** 2023-02-06

**Authors:** Zhijiang Hou, Jianyong Liu, Ming Cai, Yanpei Liu, Meiyan Zhang, Ling Wang, Wengao Yang, Bizhi Huang

**Affiliations:** 1 Institute of Alpine Economic Plant, Yunnan Academy of Agricultural Sciences, Lijiang, 674199 China; 2 Yunnan Academy of Grassland and Animal Science, Kunming, 650212 China

**Keywords:** Biotechnology, Plant sciences

## Abstract

With increasing acreage of cash crops, the use of their by-products as supplements for livestock feed becomes an important factor. Marigold (*Tagetes erecta* L.) account for more than half of the world’s loose flower production. However, there is no precedent for the abundantly available marigold crop residue (MCR) being used as feed in agricultural production, probably because of its strong pungent taste. This study aimed to evaluate the biotransformation of the volatile organic compounds (VOCs) of MCR by mixed ensilage and assess its palatability by cattle. Caryophyllene, the most prevalent VOC in MCR, decreased by 29.11% (*P* < 0.05), 38.85% (*P* < 0.05), 37.15% (*P* < 0.05), and 28.36% (*P* < 0.05) ensilage with corn meal (CM), bran (BR), crop corn (CC), and straw (ST), respectively. The acetic acid content increased by 686.05% (*P* < 0.05), 1337.21% (*P* < 0.05), 1244.19% (*P* < 0.05), and 1795.34% (*P* < 0.05) after mixed ensilage with CM, BR, CC, and ST, respectively. The total amount of alcoholic VOCs followed an overall increasing trend during mixed storage and 10 new alcohols were obtained. Over seven days, feed intake of mixed ensilage MCR by cattle differed significantly (*P* < 0.05) among treatments compared with MCR and was highest in MCRCM. Combined with palatability trials, the best MCR feed intake was achieved with MCRCM. The findings shed light on how feed odor can be improved and how degradation of terpenes can be enhanced in practical applications by mixed ensilage.

## Introduction

Lack of available roughage is of key interest to farmers and farming enterprises. To increase livestock production in the face of grassland degradation and feed crop area reduction, farmers are seeking alternative feedstuffs. With increasing acreage of cash crops, the use cash crop by-products as supplements for livestock becomes an important factor. In recent years, such cash crop by-products have been effectively adopted in animal husbandry as a replacement strategy of conventional forage, which also meets waste recycling needs as the disposal of such by-products is costly^1^.

Marigold (*Tagetes erecta* L.) is one of the most widely cultivated commercial flower crops in the world, accounting for more than half of the world’s loose flower production^2^. The high value of marigold production lies in the lutein extracted from flowers, the price of which has remained high. The rest of the plant, i.e., its stems and leaves, which are referred to as marigold crop residue (MCR), can reach around 20–30 t per hectare and are mostly dumped or burned. In fact, MCR is a nutritious feed because of its high contents of crude protein (26.53% in stem and 6.97% in leaf) and crude fat (nearly 5% in stem and nearly 2.5% in leaf), as well as its richness in amino acids and relatively low crude fiber content^3^. These nutritional traits together with extensive resource availability highlight MCR as a potential quality forage source for livestock. However, to our knowledge, there is no precedent for MCR being used as feed in agricultural production, probably because of its strong pungent taste. Research on the volatile substances present in marigold flowers and leaves has shown that various terpenoids produce terpenes of volatile organic compounds (VOCs) which are strongly rejected by cattle^4–6^. Likely, the presence of VOCs in specific terpenes affects the palatability and flavor of MCR.

To overcome such rejection by cattle, ensiling is promising as it increases palatability^7^; moreover, ensiling also preserves perishable wet biomass for extended periods^8^ and decreases the loss of nutrients^9^. Ensilage can greatly change the VOCs in forage, which has been shown for corn, alfalfa, red clover, and small grain silage^10–16^. More importantly, ensilage can greatly influence the palatability of the final forage, and is especially known to remove undesirable flavors. However, considering the role of silage in VOC production, much less information is currently available on the effects of ensilage on the biodegradation of terpenes.

Our previous study showed that during ensilage processing under laboratory conditions, lactic acid bacteria (LAB) remarkably reduced the terpene content in MCR^17^. However, the low level of water soluble carbohydrates (WSCs), the high water content in MCR, and the high cost of LAB currently limit the application of MCR in practice. Recent research showed that mixed ensilage may be a feasible method to improve silage quality compared with sole ensilage^18,19^. Corn meal (CM), bran (BR), straw (ST), and crop corn (CC) are not only easily obtainable but are also commonly used additives in mixed silage. These additives are often used to overcome problems associated with low carbohydrate content, as well as insufficiencies of LAB and water contents in sole ensilage. However, the effect of biodegradation or biotransformation of terpenes and other VOCs in mixed ensilage of MCR with CM, BR, ST, or CC must be evaluated before it can be used.

It is important to note that when ruminants approach an unfamiliar ‘putative forage’, they always employ their olfactory sense as a first gauge for edibility^20^. Although VOCs in forage can be identified and quantified using instrumental analyses, their usability, especially their acceptance level by livestock, cannot be verified with such analytic methods. Therefore, palatability trials for novel forage types that test the achieved VOCs biotransformation effect are key when using MCR as forage.

The aims of this study were to explore the VOC profile of MCR ensilage with different additives. Particular focus was placed on evaluating the biodegradation and biotransformation of the main terpenes, and to associate the effect of mixed ensilage of MCR with the palatability of the feed measured in beef cattle. The ultimate goal was to promote MCR as ruminant feed.

## Materials and methods

### Preparation of experimental silages

MCR, ST, and CC were collected from Tengchong City, Yunnan Province, China. MCR was stubbled at 5 cm over ground after harvesting the last batch of commercial flowers, and CC was harvested after milk-ripening stage. MCR and CC were air-dried to about 75% water content, ST was air-dried, and CM and BR were purchased from the local market. Before ensiling, the harvested materials were chopped to 2–5 cm using a crop chopper (model 9Z-6A, Sida Mechinery Company, Hangzhou, China). Five mixed treatments were applied: 100%MCR, 90%MCR + 10%ST (MCRST), 90%MCR + 10%CC (MCRCC), 90%MCR + 10%CM (MCRCM), and 90%MCR + 10%BR (MCRBR) (based on fresh mater). Mixed silage samples for each treatment were compacted, degassed, and sealed into plastic bags by hand, and each bag (weighing 20 ± 0.5 kg) was prepared in three replicates and stored at room temperature (15–25 ℃). After mixing ensilage for 50 days, samples were taken from each treatment and frozen at − 20 ℃ prior to analysis of VOCs.

### Experimental conditions for solid-phase microextraction

Silage samples (3 g) per treatment were placed into a crimp-top headspace vial (Agilent). After samples were melted, they were heated in a water bath at 60 °C to achieve balance of aromatic substances in the extraction vial. After 5 min of equilibrium, the aged extraction head was inserted and the sample was extracted at 60 °C for 30 min for gas chromatography–mass spectrometry (GC–MS) analysis.

### GC–MS analysis

VOCs in samples were analyzed by TRACE1310/ISQ7000 mass selective detector (ThermoFisher) with TG-5MS column (30 m × 0.25 mm × 0.25 µm; ThermoFisher). The injection was set to splitless mode for 5 min at 250 °C at a helium flow rate of 1.0 mL/min. The temperature program used 40 °C for 2 min then heating by 4 °C/min to 160 °C for 4 min and finally, to 250 °C at 15 °C/min which was maintained for 2 min. The temperature of the inlet pipe was set to 250 °C and the temperature of the ion source was set to 230 °C. The MS detector used in positron ionization (EI+) mode of 70 eV and the mass scanning range was 35–450 amu (m/z). Preliminary identification was conducted with mass spectrometry data deposited in the National Institute of Standards and Technology database (NIST 11). Identification was further achieved based on the possible percentages of the three candidate components provided by GC–MS. The relative content of each compound was calculated by the peak area normalization method where the total percentage of the peak area represents the sum of the peak areas of all identified compounds.

### Palatability trials

This experiment conformed to the Animal Welfare and Ethical Standards of the People’s Republic of China (GB14925). Research was conducted at Tengchong Hengyi Dongshan Agriculture Co., Ltd., Yunnan province, China. To determine voluntary intake of MCR, 40 18-month-old Simmental crossbred progeny (male) were distributed throughout a completely randomized experimental design with five treatments and eight replicates, and each treatment of four cattle was arranged in a pen. Animals were individually offered diets which consisted of five treatments where whole corn silage was replaced by MCR, MCRST, MCRCC, MCRCM, MCRBR, and total mixed rations (TMR), which were formulated according to United States National Research Council (NRC (2001)) and the nutrient level was basically the same.

Feed preference for mixed ensilage of MCR or a control diet was measured by weighing the animals’ intake during 30 min at the same time each morning for seven consecutive days. Each afternoon, animals were given standard TMR in addition to reach the average daily intake as measured before the experiment. TMR feeding and residual amounts were recorded daily during the test. The pen and group of each cattle remained the same throughout the experiment, and all animals had access to clean water ad libitum.

### Statistical analysis

SPSS 19.0 Statistical software was used for variance analysis, and Duncan’s test was used for multiple comparisons (*P* = 0.05). Origin 7.5 was used for mapping.

### Ethics declarations

All protocols used in the study were approved by the Animal Welfare and Ethical Standards of the People’s Republic of China (GB14925). All methods were carried out in accordance with relevant guidelines and regulations. All procedures described in this study were carried out in compliance with the ARRIVE guidelines.

## Results

### Effect of mixed ensilage on VOCs of terpenes

MCR contained 22 kinds of terpenes, accounting for 63.5% of the total VOCs. After 50 days of mixed ensiling, the quality and quantity of terpenes changed greatly. The original six terpenes disappeared completely and certain terpenes declined noticeably. The levels of caryophyllene, which accounted for 14.67% of the total VOCs, decreased by 29.11% (*P* < 0.05), 38.85% (*P* < 0.05), 37.15% (*P* < 0.05), and 28.36% (*P* < 0.05) when ensilaged with CM, BR, CC, and ST, respectively. Another main VOC, terpinolene, accounted for 8.24% of total VOCs, and decreased by 21.84% (*P* < 0.05), 13.35% (*P* < 0.05), 34.77% (*P* < 0.05), and 34.05% (*P* < 0.05) after mixed ensilage with CM, BR, CC, and ST, respectively. After mixed ensilage, six terpenes including (+)-α-pinene disappeared completely (Table 1).

**Table 1:** Effects of mixed ensilage on the VOCs of terpenes in MCR.

No	Compounds	CAS	%				
			MCR	MCRCM	MCRBR	MCRCC	MCRST
1	(+)-α-Pinene	7785-70-8	0.19 ± 0.02	ND	ND	ND	ND
2	Cyclone	508-32-7	0.17 ± 0	ND	ND	ND	ND
3	Linalool	78-70-6	0.57 ± 0.01b	0.95 ± 0.05a	0.64 ± 0.01b	0.97 ± 0.1a	0.92 ± 0.06a
4	Terpinen-4-ol	562-74-3	0.47 ± 0.11b	ND	ND	ND	0.73 ± 0.07a
5	Copaene	3856-25-5	0.08 ± 0.04	ND	ND	ND	ND
6	beta-Elemene	515-13-9	0.18 ± 0.11a	2.9 ± 0.01b	2.4 ± 0.28c	2.13 ± 0.08c	2.28 ± 0.13c
7	(-)-alpha-Gurjunene	489-40-7	0.79 ± 0.04a	0.7 ± 0.05a	0.45 ± 0.03b	ND	0.69 ± 0.03a
8	Caryophyllene	87-44-5	14.67 ± 0.45a	10.40 ± 0.36b	8.97 ± 0.47c	9.22 ± 0.64bc	10.51 ± 0.36b
9	cis-a-Bergamotene	18252-46-5	0.47 ± 0.02ab	0.51 ± 0.02a	0.3 ± 0.01cd	0.25 ± 0.01d	0.38 ± 0.06bc
10	cis-β-Farnesene	28973-97-9	9.46 ± 0.18a	8.27 ± 1.03ab	8.54 ± 0.28ab	6.64 ± 0.5b	7.72 ± 0.56ab
11	2-Epi-trans-β-caryophyllene	68832-35-9	0.24 ± 0.03	ND	ND	ND	ND
12	(1e,4e)-Germacrene b	15423-57-1	0.9 ± 0.03	ND	ND	ND	ND
13	(1s,2e,6e,10r)-3,7,11,11-Tetramethylbicyclo[8.1.0]undeca-2,6-diene	24703-35-3	0.6 ± 0.06c	ND	1.62 ± 0.12a	1.21 ± 0.02b	ND
14	ß-Longipinene	41432-70-6	0.24 ± 0.09b	ND	ND	ND	ND
15	2-Carene	554-61-0	0.17 ± 0.02A	ND	ND	0.19 ± 0.01	ND
16	Naphthalene, 1,2,3,5,6,7,8,8a-octahydro-1,8a-dimethyl-7-(1-methylethenyl)-, [1s-(1a,7a,8aa)]-	10219-75-7	0.09 ± 0.09b	0.27 ± 0.1a	0.15 ± 0.01ab	ND	ND
17	(-)-β-Pinene	18172-67-3	3.01 ± 0.27a	0.64 ± 0.11b	1.03 ± 0.04b	1.03 ± 0.38b	0.62 ± 0.08b
18	Terpinolene	586-62-9	8.24 ± 0.25a	6.44 ± 0.18bc	7.14 ± 0.11b	5.34 ± 0.16c	5.4 ± 0.33c
19	d-Limonene	5989-27-5	5.56 ± 0.14a	2.71 ± 0.15c	4.58 ± 0.14b	2.21 ± 0.15d	1.79 ± 0.21d
20	ß-Ocimene	13877-91-3	4.92 ± 0.31a	3.08 ± 0.27b	4.66 ± 0.21a	2.69 ± 0.17c	1.78 ± 0.11d
21	Piperitone	89-81-6	12.17 ± 0.11a	10.09 ± 0.79b	10.16 ± 0.25b	11.2 ± 0.35ab	10.41 ± 0.67ab
22	Sabinene	3387-41-5	0.31 ± 0.06b	3.52 ± 0.26a	3.00 ± 0.31a	3.66 ± 0.41a	3.42 ± 0.27a
23	Globulol	489-41-8	ND	ND	ND	0.16 ± 0.02	ND
24	Nerolidol	142-50-7	ND	0.14 ± 0.01b	0.18 ± 0.02b	0.2 ± 0.01b	0.26 ± 0.03a
25	(-)-allo-Aromade0rene	25246-27-9	ND	ND	ND	0.19 ± 0.01	ND
26	(+)-Ledene	21747-46-6	ND	1.05 ± 0.21b	3.11 ± 0.18a	3.26 ± 0.05a	ND
27	Bicyclo[7.2.0]u0ec-4-ene, 4,11,11-trimethyl-8-methylene-,[1R-(1R*,4Z,9S*)]-	118-65-0	ND	0.41 ± 0.04a	0.15 ± 0.04b	0.17 ± 0.02b	ND
28	(1R,2S,6S,7S,8S)-8-Isopropyl-1-methyl-3-methylenetricyclo[4.4.0.02,7]decane-rel-	18252-44-3	ND	ND	0.53 ± 0.06a	ND	0.2 ± 0.02b
29	α-Himachalene	3853-83-6	ND	0.23 ± 0.04	ND	ND	ND
30	α-Elemene	63929-10-2	ND	2.06 ± 0.35	ND	ND	ND
31	3,8,8-Trimethyl-6-methyleneoctahydro-1H-3a,7-methanoazulene	79120-98-2	ND	0.94 ± 0.09a	0.56 ± 0.02b	ND	ND
32	(-)-Germacrene-D	317819-80-0	ND	1.41 ± 0.28a	0.51 ± 0.05b	ND	0.2 ± 0.01bc
33	(-)-Terpinen-4-ol	20126-76-5	ND	6.97 ± 1.08c	4.72 ± 1.52d	9.05 ± 1.1a	8.03 ± 1.32b
34	1H-Cycloprop[e]azulene, decahydro-1,1,7-trimethyl-4-methylene-	72747-25-2	ND	0.63 ± 0.06a	0.48 ± 0.02a	ND	0.3 ± 0.08b
35	diosphenol	490-03-9	ND	0.24 ± 0.01a	0.25 ± 0.01a	0.17 ± 0.02b	0.28 ± 0.02a
36	Naphthalene, 1,2,3,5,6,7,8,8a-octahydro-1,8a-dimethyl-7-(1-methylethenyl)-, [1S-(1a,7a,8aa)]-	10219-75-7	ND	0.27 ± 0.1a	0.15 ± 0.01ab	ND	ND
37	α-Thujone	62181-90-2	ND	1.52 ± 0.31a	ND	1.43 ± 0.03a	ND
38	(5R)-5-Isopropenyl-2-methyl-2-cyclohexen-1-ol	99-48-9	ND	ND	ND	0.53 ± 0.05	ND
39	α-Longipinene	5989-8-2	ND	0.72 ± 0.25a	0.44 ± 0.13ab	ND	ND
40	1,5,5-Trimethyl-6-methylidenecyclohexene	514-95-4	ND	ND	0.14 ± 0.01	ND	ND
41	(+)-alpha-Terpineol	7785-53-7	ND	1.01 ± 0.03a	ND	0.92 ± 0.24a	ND
42	8alpha-Hydroxy-alpha-gurjunene	70206-70-1	ND	ND	ND	ND	0.77 ± 0.01
Total			63.5	68.08	64.86	62.72	56.69

CAS, chemical abstract service; ND, not detected; experiments were performed in triplicate and shown are the means ± S.D., different lowercase letters correspond to a significant difference at the 0.05 level (Duncan's new multiple range test). The same as Table 2.

With progressing mixed ensilage, certain original terpenes disappeared, and 20 terpenes emerged, including eight terpenes that were only present in a single treatment, suggesting that these may have been contained in the additive of each treatment. However, among the newly emerged terpenes, three were simultaneously found in all treatments, suggesting that their formation may have been a bioconversion from the original terpenes.

### Effect of mixed ensiling on VOCs of alcohol and acid changes

Fresh MCR contain only one acid—acetic acid. Its contents increased by 686.05% (*P* < 0.05), 1337.21% (*P* < 0.05), 1244.19% (*P* < 0.05), and 1795.34% (*P* < 0.05) after mixed ensilage with CM, BR, CC, and ST, respectively. Five kinds of acids that emerged during the mixed ensilage process appeared in different treatments among which lactic acid was only found in MCRCC (Table 2).

**Table 2:** Effects of mixed ensilage on the VOCs of alcohols and acids in MCR.

No	Compounds	CAS	%				
			MCR	MCRCM	MCRBR	MCRCC	MCRST
Acids							
1	Acetic acid	64-19-7	0.57 ± 0.01d	3.38 ± 1.31c	6.18 ± 2.53b	5.78 ± 0.92b	8.15 ± 1.01a
2	Lactic acid	50-21-5	ND	ND	ND	2.34 ± 1	ND
3	n-Heptanoic acid	142-62-1	ND	ND	ND	0.28 ± 0	ND
4	Octanoic acid	124-07-2	ND	0.26 ± 0.01	ND	ND	ND
5	(E)-3-hexenoic acid	1577-18-0	ND	ND	ND	0.27 ± 0.01	ND
6	Cyclohexanebutyric acid	4441-63-8	ND	ND	ND	0.31 ± 0.05	ND
Alcohols							
7	Phenylethyl alcohol	60-12-8	0.49 ± 0.27c	0.86 ± 0.14b	1.45 ± 0.04a	0.72 ± 0.11b	1.58 ± 0.05a
8	1-Propanol	71-23-8	ND	0.71 ± 0.44a	0 ± 0	ND	ND
9	Ethanol	64-17-5	ND	0.6 ± 0.47a	0.27 ± 0.04a	ND	ND
10	(±)-2-Butanol	78-92-2	ND	ND	ND	ND	2.89 ± 0.78a
11	(3-Methyl-oxiran-2-yl)-methanol	872-38-8	ND	ND	ND	ND	3.35 ± 0.37
12	Leaf alcohol	928-96-1	ND	ND	ND	1.74 ± 0.18	ND
13	Acetic acid,hex-4-en-1-ol	72237-36-6	ND	0.35 ± 0.04	ND	ND	ND
14	Benzyl alcohol	100-51-6	ND	0.43 ± 0.11a	0.29 ± 0.06ab	0 ± 0	0.2 ± 0.01bc
15	(E)-3-phenylbut-2-enal	1196-67-4	ND	0 ± 0	0.16 ± 0.02b	0.28 ± 0.07a	ND
16	Hotrienol	20053-88-7	ND	ND	0.26 ± 0.07	ND	ND
17	Leaf alcohol	928-96-1	1.42 ± 0.19	ND	ND	ND	ND
18	trans-Chrysanthenol	38043-83-3	0.15 ± 0.01	ND	ND	ND	ND
19	1-(2,6,6-Trimethyl-1,3-cyclohexadienyl)ethane	102676-97-1	ND	ND	ND	0.33 ± 0.12	ND
20	1-Hexanol	111-27-3	0.42 ± 0.07	ND	ND	ND	ND

The alcohol levels of MCR and mixed ensilage of MCR are shown in Table 2. After 50 days of ensilage, the total amount of alcoholic VOCs showed an overall increasing trend during the mixed storage process and 10 new alcohols had formed. Among these, (±)-2-butanol and 3-Methyl-oxiran-2-yl)-methanol produced by MCRST contents accounted for 2.89% and 3.35% of total VOCs, respectively. Three alcohols disappeared during mixed storage with the exception of phenylethyl alcohol.

### Effect of mixed ensiling on VOCs of other VOC changes

Ensilage not only transformed terpenes, alcohols, and acids in MCR, it also changed the types and contents of other VOCs (Table 3). After 50 days of ensilage, the total VOCs of aldehydes, benzenes, alkenes, and miscellaneous compounds decreased by 50.22%, 47.37%, 3.3%, and 25.29%, while the contents of esters, phenols, alkanes, and furan increased by 54.07%, 233.33%, 19.46%, 69.64%, and 173.56%, respectively.

**Table 3:** Effects of mixed ensilage on the contents of VOCs in MCR.

No	Compounds	%				
		CK	MCRCM	MCRBR	MCRCC	MCRST
1	Aldehydes	11.35	3.99	5.51	6.94	5.64
2	Phenols	0.48	0.71	0.77	1.25	1.6
3	Esters	3.81	5.43	5.57	6.02	5.87
4	Alkanes	4.83	6.8	7.01	7.65	5.77
5	Alkenes	2.12	0.47	0.64	0.41	2.05
6	Benzenes	1.33	1.1	0.99	1.03	0.7
7	Furans	0.56	1.2	1.02	0.94	0.95
8	Miscellaneous	0.87	0.68	0.62	0.64	0.65

### Palatability trials

**Figure 1:**
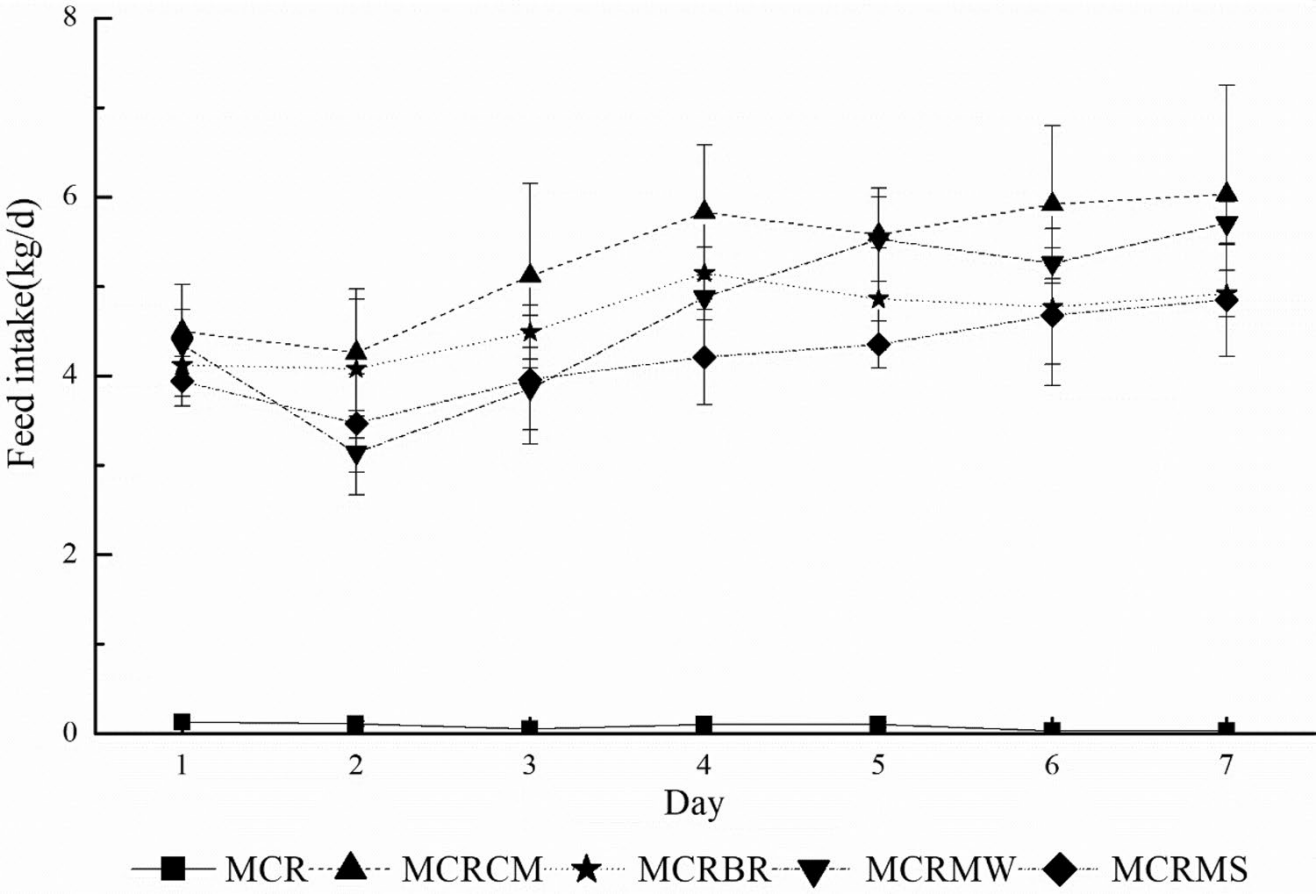
Mean intake of treated marigold crop residue (MCR) by cattle during a 30-min interval for seven consecutive days.

Feed intake of mixed ensilage MCR over seven days is reported in Fig. 1, which shows the average fresh material intake of cattle. As expected, all mixed silage intake levels of cattle significantly (*P* < 0.05) differed among treatments compared with MCR. Intake levels were highest in MCRCM, followed by MCRCC, MCRBR, and MCRST presenting a clear preference gradient. This indicates that the intake differences between the most and the least preferred MCR combinations were considerable, which is mainly affected by terpenes.

## Discussion

Gao et al.^21^ found that terpenes are the major components in the VOCs of marigold flowers. The cis-β-farnesene, caryophyllene, terpinolene, and piperitone obtained in this study agree with the research of Martha et al.^5^ and Ogunwande and Olawore^6^ who identified these terpenes as the major components of the essential oil in marigold leaves and flowers. These results suggest that mixed ensilage mediated the degradation of certain terpenes, which is consistent with previous findings. For example, Figueiredo et al.^10^ also found that certain terpenes in red clover forages were significantly reduced after ensilage. The reason for this reduction of terpenes is not fully understood, but may involve oxidation to secondary products, isomerization, and/or interconversion of certain monoterpenols, as well as ester conversion or glycoside hydrolysis^22^. It should be noted that not all original terpene levels decreased, the levels of a few terpenes that accounted for a small amount of total VOCs even increased. While ensilage reduces the total terpene content in red clover, the levels of a few terpenes increased^10^, and the total terpene content either decreased or increased with fermentation of different fruit juices^23,24^. Information regarding terpene biodegradation is scarce at present and underlying the mechanism remains unclear.

Although terpenes formally consist of a single biosynthetic unit, in fact, they can be biotransformed by mechanisms such as dehydrogenation, hydration, isomerization, conjugation, reduction, oxidation, β-oxidation, and decarboxylation, indicating that a variety of structures can be produced^22^. (-)-Terpinen-4-ol was a new and considerably produced terpene which accounted for 6.97% (*P* < 0.05), 4.72% (*P* < 0.05), 9.05% (*P* < 0.05), and 8.03% (*P* < 0.05) in mixed ensilages with CM, BR, CC, and ST, respectively. However, a previous study on MCR ensilaged with LAB at different ensilage stages did not find this terpene^17^, and the reason for the emergence of such a large amount of terpene during the mixed ensilage process required further study.

In fact, a range of microorganisms promote the biotransformation of terpenes during ensilage, including bacteria, fungi, and yeasts. These various microorganism-facilitated biotransformation reactions convert original terpenes into new ones and other substances. Although mixed ensilage can modulate terpene changes, it is very difficult to infer complex relationships from a final quantity change or kinetic change of terpenes, as many other compounds can also interact with terpenes or can affect their metabolic behavior^22,25^. Thus, mixed ensilage can not only degrade terpenes, it can also produce new terpene metabolites. Overall, 14, 12, 10, and 7 new terpenes were produced in MCRCM, MCRBR, MCRCC, and MCRST, respectively. These terpenes accounted for a relatively small proportion except for (-)-Terpinen-4-ol. Mixed ensilage significantly decreased the contents of major terpenes in MCR in all treatments.

As one of the most important organic acids in silage, acetic acid affects the quality of silage, and is known to emit an acidic odor^26^. The accumulation of acetic acid depends on the substrate supply of the starter and the sugar metabolism^27^. In the fat metabolism in silage, fermentation can degrade fatty acids and produce short-chain fatty acids such as acetic acid, butyric acid, and caprylic acid. As more acids may be produced in other silages and food fermentation reactions, this experiment yielded less acids, and lactic acid only appears in MCRCC. This may be related to the large number of LAB attached to the whole corn plant^17^, while other treatments may have used other microbiological fermentation processes.

The alcohol changes are consistent with the results of Figueiredo et al.^10^ who also found that the alcohol level in red clover changed after ensilage and part of all alcohols disappeared. Alcohol levels varied greatly among CC, alfalfa, grain, and red clover silage^10,15,28^. Recent studies have shown that the ethanol content in CC silage is as high as 70% of the total VOCs^13,29^, but the present study only detected very low ethanol contents in MCRCM and MCRBR. With the exception of ethanol, data on alcohols in silage are rare. It may be produced by catabolism of amino acids or reduction of aldehydes and ketones^30^. Forages with high dry matter content produce higher ethanol content because of their high fermentable carbohydrate content. In contrast, low carbohydrate leguminous forages tend not to produce such a high ethanol content during ensilage^10,16^. This may be the main reason why the low carbohydrate MCR ensiling resulted in less ethanol production. However, silage quality does not manifest in the production of a large amount of ethanol, as this has adverse effects on the environment and the animals themselves^15,28,31^.

Other VOCs might strongly affect animal acceptance of forage even at lower concentrations^32^. In this study, it is therefore impossible to clarify the relationship between MCR silage and VOC biotransformation and the effects of the various VOCs observed can also not be distinguished. Therefore, more research must be carried out on this specific relationship.

Intake was negatively correlated with dietary terpene concentration^33,34^, and ruminants limited excessive terpene intake by extending their feeding time^35^. In addition, mixed MCR ensilage also caused significant differences in feed intake. However, the correlation between the feed intake trial results and the identification and quantitation of VOCs poses a difficult problem for the following reasons: (a) In domestic herbivores, feed intake is clearly affected by previous feeding experience, and memory is elicited by familiar sensorial perceptions^36–38^. The cattle in this experiment had feeding experience of whole corn silage and concentrate containing CM and BR, but they had no straw feeding experience at all. Thus, previous experience may be more important than innate preference compared to specific VOCs. (b) The flavors of feeds are synthesized by large amounts of VOCs. Even though VOCs were identified and quantified in all treatments, it was difficult to determine which VOCs at which concentrations affected palatability in cattle.

As similar effects were observed in previous studies^36,37^, the overall trend of lower intake over the first one or two days was not surprising (Fig. 1). The palatability trial results showed that treatments in the first day or two portrayed significant neophobia, after which, a rapid learning process could be observed, which may be related to strong post-ingestive effects attributable to feed characteristics^20,39^. These results suggest MCR was not acceptable to cattle at any time. However, other treatments strongly increased feed intake after a day or two, which to some extent explains why the mixed MCR silage not only had a smell that differed from the original and was more acceptable for cattle, the satiation threshold for terpenes was also not reached. This resulted in the sensory feedback of cattle that reduced feed intake.

## Conclusions

This work presents the first study to the biotransformation of VOCs in marigold mixed silage and its palatability in ruminants. Mixed ensilage affected the quantitative and qualitative profile of VOCs in MCR. The main terpenes decreased significantly, while the alcohols and acids increased significantly. However, it is impossible to infer which treatment achieved the best effect on VOC changes in MCR. Combined with palatability trials, the best MCR feed intake was achieved with MCRCM. The findings of this study shed light on how feed odor can be improved and how degradation of terpenes can be enhanced in practical applications by mixed ensilage.

## Data Availability

All data included in this study are available upon request by contact with the corresponding author.
